# Towards a biomarker for acute arterial thrombosis using complete blood count and white blood cell differential parameters in mice

**DOI:** 10.1038/s41598-023-31122-9

**Published:** 2023-03-10

**Authors:** Hee Jeong Jang, Dawid Schellingerhout, Jiwon Kim, Jinyong Chung, Dong-Eog Kim

**Affiliations:** 1 Department of Medical Biotechnology, Dongguk University, Seoul, Republic of Korea; 2 Molecular Imaging and Neurovascular Research Laboratory, Department of Neurology, Dongguk University College of Medicine, Dongguk University Ilsan Hospital, Goyang, Republic of Korea; 3 Departments of Diagnostic Radiology and Cancer Systems Imaging, University of Texas M. D. Anderson Cancer Center, Houston, TX USA; 4 Department of Neurology, Dongguk University College of Medicine, Seoul, Republic of Korea; 5 Medical Science Research Center, Dongguk University Medical Center, Goyang, Republic of Korea; 6 National Priority Research Center for Stroke, Goyang, Republic of Korea

**Keywords:** Biomarkers, Diagnostic markers

## Abstract

There is no blood biomarker diagnostic of arterial thrombosis. We investigated if arterial thrombosis per se was associated with alterations in complete blood count (CBC) and white blood cell (WBC) differential count in mice. Twelve-week-old C57Bl/6 mice were used for FeCl_3_-mediated carotid thrombosis (n = 72), sham-operation (n = 79), or non-operation (n = 26). Monocyte count (/µL) at 30-min after thrombosis (median 160 [interquartile range 140–280]) was ~ 1.3-fold higher than at 30-min after sham-operation (120 [77.5–170]), and twofold higher than in non-operated mice (80 [47.5–92.5]). At day-1 and -4 post-thrombosis, compared with 30-min, monocyte count decreased by about 6% and 28% to 150 [100–200] and 115 [100–127.5], which however were about 2.1-fold and 1.9-fold higher than in sham-operated mice (70 [50–100] and 60 [30–75], respectively). Lymphocyte counts (/µL) at 1- and 4-days after thrombosis (mean ± SD; 3513 ± 912 and 2590 ± 860) were ~ 38% and ~ 54% lower than those in the sham-operated mice (5630 ± 1602 and 5596 ± 1437, respectively), and ~ 39% and ~ 55% lower than those in non-operated mice (5791 ± 1344). Post-thrombosis monocyte-lymphocyte-ratio (MLR) was substantially higher at all three time-points (0.050 ± 0.02, 0.046 ± 0.025, and 0.050 ± 0.02) vs. sham (0.003 ± 0.021, 0.013 ± 0.004, and 0.010 ± 0.004). MLR was 0.013 ± 0.005 in non-operated mice. This is the first report on acute arterial thrombosis-related alterations in CBC and WBC differential parameters.

## Introduction

There is no specific blood biomarker for de novo arterial thrombosis at an early phase; knowledge of such a biomarker would help predict the occurrence of stroke or myocardial infarction, and perhaps help in making treatment decisions. White blood cell (WBC) count, neutrophil lymphocyte ratio (NLR), monocyte lymphocyte ratio (MLR), and platelet lymphocyte ratio (PLR) have all been reported to be prognosticators of thrombotic cardiovascular events^1^. A previous clinical study showed that NLR could predict the presence of atrial fibrillation (AF)-related cardiac thrombus^2^, which is a common cause of embolic and not in situ occlusion of cerebral arteries. According to investigators from the Copenhagen General Population Study who retrospectively reviewed hospital records, high platelet counts (> 95th percentile, 398 × 10^3^/µL) were associated with a 1.8-fold increased risk of ischemic stroke^3^. Recently, NLR and PLR were shown to be associated with recurrent ischemic stroke in patients with embolic stroke of undetermined source, even after adjustment for vascular risk factors, newly diagnosed AF, and left atrial volume index^4^.

To date, it is not known whether arterial thrombosis per se could alter complete blood count (CBC), WBC differential count, NLR, MLR, and PLR. This question is difficult to study in patients, because of the difficulty of obtaining blood samples close enough to the thrombotic event (and uncertainty regarding its timing), the effects of many confounders (such as organ damage and severity of thrombosis), and a lack of appropriate controls. We chose to investigate this question in the well-known in vivo model of FeCl_3_ (Ferric chloride)-mediated in situ carotid thrombosis by measuring alterations in the CBC-related parameters.

## Results

### There was no thrombosis-related mortality, cortisol elevation, or anemia

**Figure 1:**
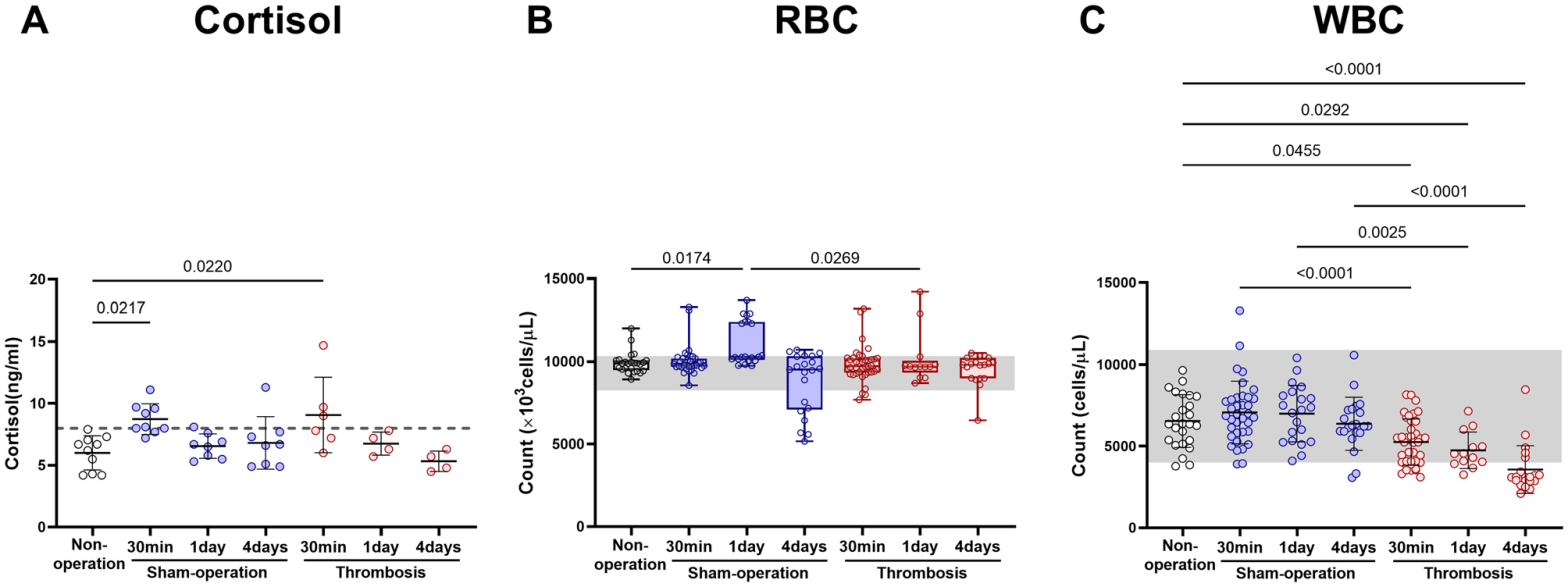
Arterial thrombosis-related alterations in serum cortisol level, complete blood count (CBC), and white blood cell (WBC) differential count. (**A**) Cortisol. (**B**) Red blood cell (RBC). (**C**) White blood cell (WBC). Scatter plots (with mean ± SD) are for cortisol levels and WBC: *p* values are from One-way ANOVA and Bonferroni's post-hoc tests. Box plots are for red blood cell count: *p* values are from Kruskal–Wallis ANOVA and Dunn’s post-hoc tests. The box covers the interquartile interval (25–75%), with the horizontal line in the box representing the median. The whiskers outside the box correspond to the minimum and maximum values. The dotted horizontal line represents an approximate reference value that was previously reported^5^. Gray shades represent reported reference value ranges^6^. Cortisol levels: Non-operation group (n = 10), Sham-operation groups (n = 9 at 30 min, n = 8 at day 1, and n = 8 at day 4), Thrombosis groups (n = 6 at 30 min, n = 4 at day 1, n = 4 at day 4). RBC and WBC counts: Non-operation group (n = 26), Sham-operation groups (n = 37 at 30 min, n = 21 at day 1, and n = 21 at day 4), and Thrombosis groups (n = 39 at 30 min, n = 13 at day 1, and n = 20 at day 4).

There was no mortality in our study. As shown in Fig. 1A, cortisol levels (reported^5^ mean reference value: about 8 ng/ml) were about 50% higher both at 30 min after thrombosis (mean ± SD; 9.07 ± 3.04) and at 30 min after sham operation (8.73 ± 1.25), compared with non-operated mice (6.00 ± 1.38). However, at days 1 and 4, there was no significant inter-group difference. Moreover, there was no post-operative anemia (Fig. 1B); red blood cell (RBC) counts (reported^6^ reference value range: 8250–10,330 × 10^3^/µL) at all three time-points in thrombosed mice did not differ significantly when compared with RBC counts at the corresponding time-points (except day 1) in sham-operated mice and with the single time-point data in non-operated mice. RBC count at 1 day after sham-operation (median [interquartile range], 10,280 [10105–12400]) was significantly but only slightly higher when compared to both post-thrombosis day 1 (9700 [9335–10045]) and non-operation (9880 [9480–10070]).

### Monocytosis was observed post-thrombosis 30 min, 1 day, and 4 days, while relative lymphopenia was observed at 1 and 4 days in a mouse model of FeCl_3_-mediated carotid thrombosis

White Blood Cell (WBC) counts (reported^6^ reference value range: 4000–10,900/µL) were about 25–44% lower at 30 min (mean ± SD, 5252 ± 1414), 1 day (4747 ± 1118), and 4 days (3570 ± 1461) after carotid thrombosis, compared with the corresponding time-points after sham operation (7038 ± 1923, 6991 ± 1733, and 6373 ± 1630; Fig. 1C). Unlike in sham-operated mice, the WBC count at each of the three time-points in thrombosed mice was lower than the WBC count (measured once) in non-operated mice (6539 ± 1617).

**Figure 2:**
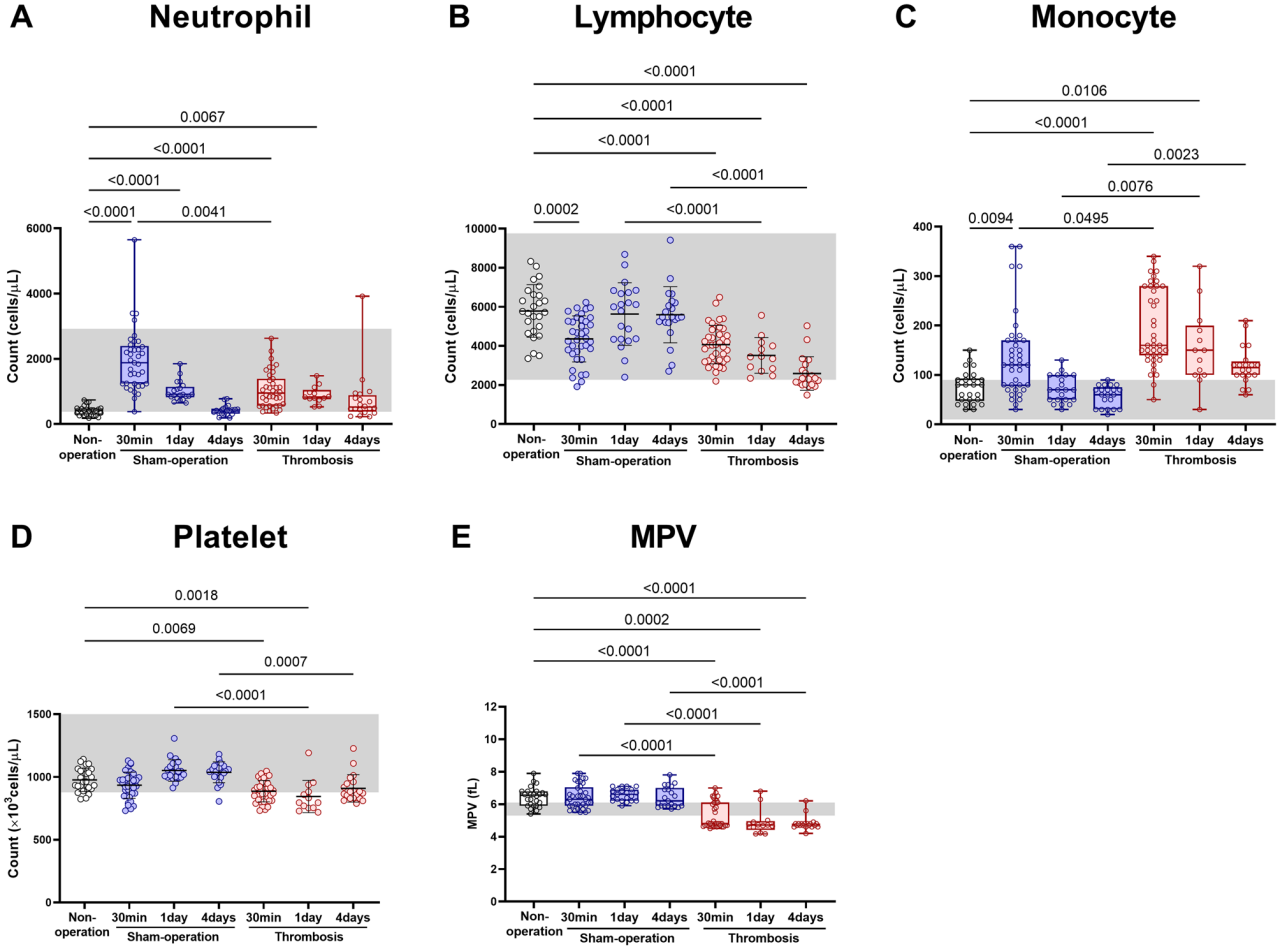
Arterial thrombosis-related alterations in complete blood count (CBC) and white blood cell (WBC) differential count. (**A**) Neutrophil. (**B**) Lymphocyte. (**C**) Monocyte. (**D**) Platelet. (**E**) Mean platelet volume (MPV). Box plots are for neutrophil count, monocyte count, and mean platelet volume (MPV): *p* values are from Kruskal–Wallis ANOVA and Dunn’s post-hoc tests. The box covers the interquartile interval (25% ~ 75%), with the horizontal line in the box representing the median. The whiskers outside the box correspond to the minimum and maximum values. Scatter plots (with mean ± SD) are for lymphocyte count and platelet count: *p* values are from One-way ANOVA and Bonferroni's post-hoc tests. Gray shades represent reported reference value ranges^6^. Non-operation group (n = 26), Sham-operation groups (n = 37 at 30 min, n = 21 at day 1, and n = 21 at day 4), and Thrombosis groups (n = 39 at 30 min, n = 13 at day 1, and n = 20 at day 4).

Neutrophil count (reported^6^ reference value range: 380–2920/µL) was about twofold higher at 30 min and 1 day after carotid thrombosis (median [interquartile range], 950 [560–1390] and 830 [760–1050], respectively) compared with non-operated mice (415 [285–483]; Fig. 2A). However, neutrophil count at 4 days after thrombosis (520 [380–890]) did not differ significantly when compared with the corresponding value in non-operated mice. In sham-operated mice, neutrophil count gradually decreased (1880 [1240–2400] at 30 min, 910 [820–1140] at day 1, and 420 [345–470] at day 4) to the level of non-operated mice. Although neutrophil count at 30 min was unexpectedly higher in sham-operated mice than in thrombosed mice, there were no significant inter-group differences at days 1 and 4.

Lymphocyte count (reported^6^ reference value range: 2270–9760/µL) was lower at 30 min after carotid thrombosis (mean ± SD, 4067 ± 982.2) and after sham operation (4361 ± 1179) by about 30% and 25%, respectively, than in non-operated mice (5791 ± 1344; Fig. 2B). Lymphocyte count decreased further (to about 51% vs. sham) by day 4 in thrombosed mice (2590 ± 860) whereas it increased to the level of non-operated mice by day 1 in sham-operated mice (5630 ± 1602).

Monocyte count (reported^6^ reference value range: 10–90/µL) was higher at 30 min after carotid thrombosis (median [interquartile range], 160 [140–280]) and at 30 min after sham operation (120 [77.5–17]), by twofold and 1.5-fold, respectively, than in non-operated mice (80 [47.5–92.5]; Fig. 2C). It was notable that the monocyte count at 30 min was about 1.3-fold higher after thrombosis than after sham operation. Thereafter, the monocyte count in sham-operated mice rapidly decreased by day 1 (to 70 [50–100], which was similar to the level of non-operated mice), while in thrombosed mice slowly and less markedly decreased by day 4 (to 115 [100–127.5]). Thus, the monocyte count at day 4 was about 1.9-fold higher in thrombosed mice than in sham-operated mice (60 [30–75]), although the monocyte counts at day 4 in both groups did not show a significant difference when compared with those values in non-operated mice.

### Relative thrombocytopenia was observed post-thrombosis 1 and 4 days, while reduction in MPV was observed at 30 min, 1 day, and 4 days after FeCl_3_-mediated carotid thrombosis in mice

Platelet count (reported^6^ reference value range: 878–2283 × 10^3^/µL) was slightly (about 9.2%) lower at 30 min after carotid thrombosis (mean ± SD, 887.2 ± 85.47), not after sham operation (934.3 ± 104.1), when compared with non-operated mice (977.1 ± 92.46; Fig. 2D). The platelet counts at 30 min in thrombosed mice and those in sham-operated mice did not differ significantly. Thereafter, in sham-operated mice, platelet count slightly (about 12.8%) increased by day 1 (1052 ± 86.1), whereas it did not change significantly over 4 days in thrombosed mice (844.7 ± 129.1 at day 1 and 909.2 ± 109.1 at day 4). Thus, the platelet counts at 1 and 4 days after thrombosis were slightly (about 20% and 12%) lower than at the corresponding time-point in sham-operated mice (1052 ± 86.1 and 1038 ± 83.04). MPVs (reported^6^ reference value range: 5.3–6.1 fL) were about 23 ~ 28% lower at all three time-points in thrombosed mice (4.8 [4.7–6.1], 4.72 [4.41–4.95] and 4.75 [4.63–4.8]) compared with the corresponding time-points in sham-operated mice (6.3 [5.9–7.05], 6.6 [6.2–6.9], and 6.2 [5.9–7]) and the single time-point in non-operated mice (6.55 [5.9–6.8]; Fig. 2E).

### NLR was high at 4 days after FeCl_3_-mediated carotid thrombosis in mice

**Figure 3:**
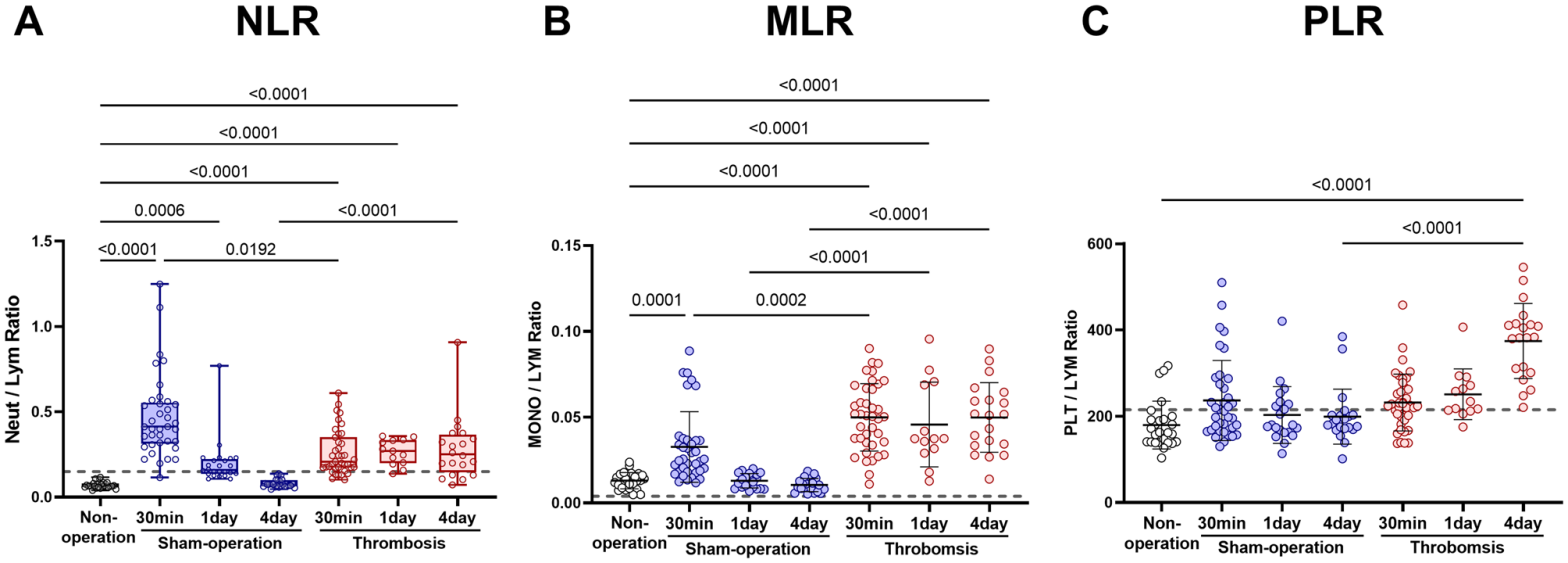
Comparison of neutrophil lymphocyte ratio (NLR), monocyte lymphocyte ratio (MLR), and platelet-lymphocyte ratio (PLR). (**A**) NLR. (**B**) MLR. (**C**) PLR. Box plots are for NLR: *p* values are from Kruskal–Wallis ANOVA and Dunn’s post-hoc tests. The box covers the interquartile interval (25–75%), with the horizontal line in the box representing the median. The whiskers outside the box correspond to the minimum and maximum values. Scatter plots (with mean ± SD) are for MLR and PLR: *p* values are from One-way ANOVA and Bonferroni's post-hoc tests. Dotted lines represent proposed reference (ratio) values, calculated by using the mean values of the reference WBC differential count^6^. Note that SDs could not be calculated because of the unavailability of the raw data. Non-operation group (n = 26), Sham-operation groups (n = 37 at 30 min, n = 21 at day 1, and n = 21 at day 4), and Thrombosis groups (n = 39 at 30 min, n = 13 at day 1, and n = 20 at day 4).

NLRs (0.15 as a reference ratio, calculated with reported^6^ reference median values of WBC differential counts) were about threefold higher at 30 min after carotid thrombosis (median [interquartile range], 0.21 [0.17–0.35]), and they were—unexpectedly—about sixfold higher at 30 min (0.41, 0.31–0.55) after sham operation, compared with non-operated mice (0.07 [0.06–0.08]; Fig. 3A). Although the NLR at 30 min was about twofold higher in sham-operated mice (vs. thrombosed mice), it rapidly decreased by day 1 to the level of 0.16 [0.14–0.22] (numerically lower vs. 0.27 [0.2–0.33] in thrombosed mice), then further decreased by day 4 to the level of 0.07 [0.06–0.1], which was very similar to the value obtained in non-operated mice. However, in thrombosed mice, the NLR remained similarly and substantially high by day 4 (0.25 [0.15–0.37]). Thus, the NLR at 4 days after thrombosis was about 3.5-fold higher than that in sham-operated mice as well as non-operated mice.

### MLR was high at 30 min, 1 day, and 4 days after FeCl_3_-mediated carotid thrombosis in mice

MLR (0.004 as a reference ratio, calculated with reported^6^ reference median values of WBC differential counts) was found to be at its lowest value in non-operated mice (mean ± SD, 0.013 ± 0.005), a higher value in sham-operated mice (0.030 ± 0.02; about 2.3-fold higher vs. non-operated mice), and its highest value in thrombosed mice at 30 min (0.050 ± 0.02; about 3.8-fold higher vs. non-operated mice and about 1.7-fold higher vs. sham-operated mice; Fig. 3B). Moreover, in sham-operated mice, MLR rapidly decreased by day 1 to 0.013 ± 0.004, which was very similar to the level of non-operated mice. However, in thrombosed mice, MLR remained similarly high by day 4 (0.046 ± 0.02 at day 1 and 0.050 ± 0.02 at day 4).

### PLR was high at 4 days after FeCl_3_-mediated carotid thrombosis in mice

PLR (215.37 as a reference ratio, calculated with reported^6^ reference median values of WBC differential counts) was high 4 days after carotid thrombosis (374.4 ± 86.87), about 1.5-fold higher compared with prior post-thrombosis time-points (231.8 ± 65.71 at 30 min and 250.8 ± 58.82 at day 1), about ~ 1.9-fold higher compared with all three time-points in sham-operated mice (236.5 ± 92.87 at 30 min and 202.6 ± 65.93 at day 1 and 199.1 ± 65.71 at day 4), and about twofold higher compared with the single time-point in non-operated mice (179.7 ± 55.51; Fig. 3C).

### Principal component analysis (PCA) further corroborated aforementioned CBC alterations due to acute arterial thrombosis

**Figure 4:**
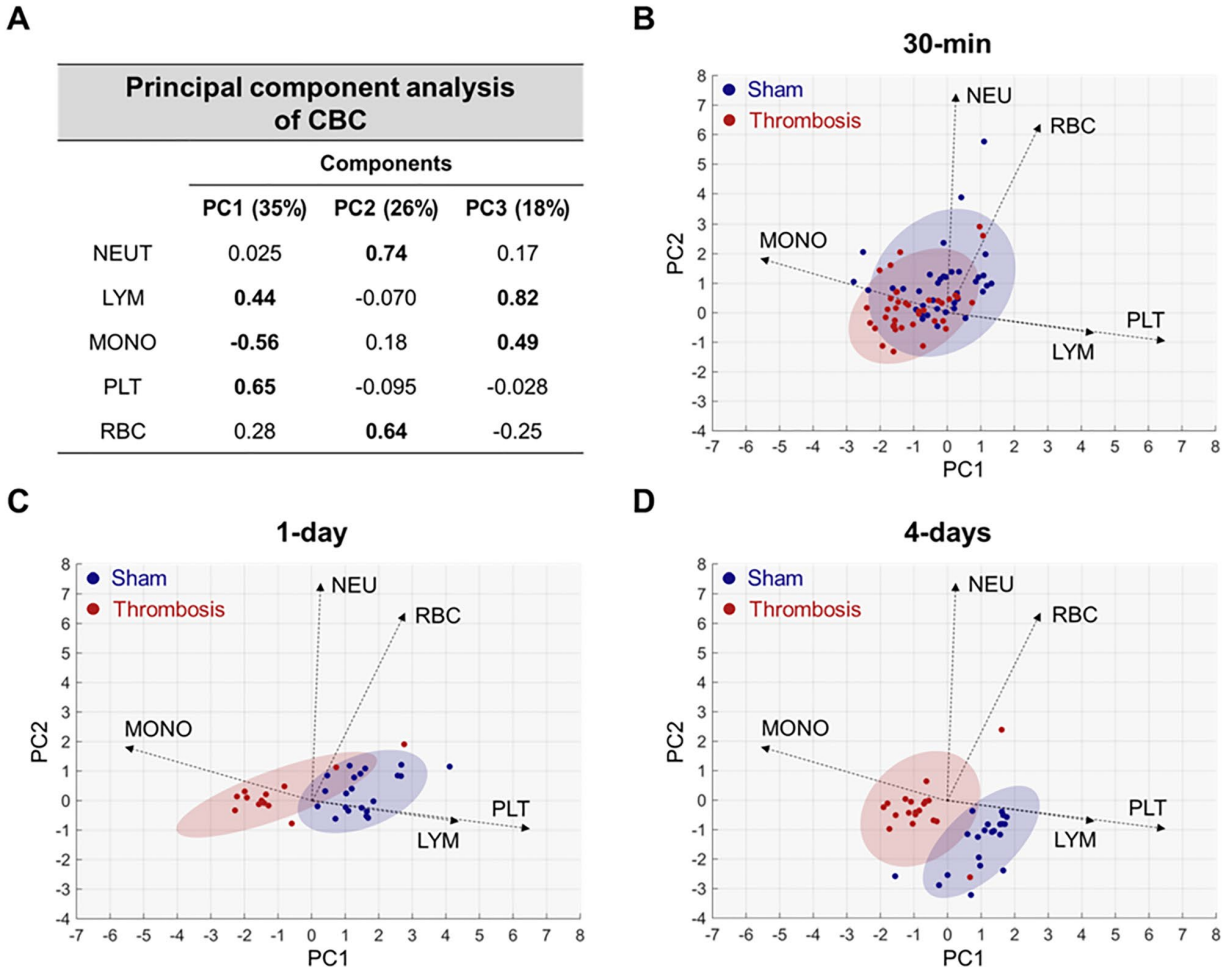
Principal component analysis (PCA) of complete blood count (CBC) parameters in sham-operated and thrombosed mice. (**A**) Loading weights of CBC parameters and explained variances (%) for the first three PCs (PC1 ~ 3). Values greater than 0.4 are bolded. (**B**) PCA biplot (on PC1 and PC2) for the samples from sham-operated (blue) and thrombosed (red) mice at 30 min. Loading vectors are presented as dotted arrows, with their sizes being 10-times higher than the loading weights for PC1 and PC2 to improve the visualization. Based on a two-dimensional normal distribution fitted to each group’s data, each shaded area covers 90% of the sample distribution of the corresponding group. (**C**) PCA biplot at day 1. (**D**) PCA biplot at day 4. PCA identifies the most important sources of variability in the collective datasets (neutrophil count [NEU], lymphocyte count [LYM], monocyte count [MONO], platelet count [PLT], and red blood cell count [RBC]) at all three time-points. PCA biplots^42^ were generated to allow for visual assessments of data groupings by plotting the samples in two dimensions using their orthogonal projections onto the first two principal components (PC1 and PC2). Loading vectors were displayed in the biplots to explain how each CBC parameter contributed to the variability in the PC space. The first two PCs explained 61% of the total variability. Platelet, monocyte, and lymphocyte counts contributed mainly to PC1, and neutrophil and RBC counts contributed mainly to PC2. Accordingly, the loading vectors of monocyte, lymphocyte, and platelet counts were approximately parallel to PC1, whereas the loading vectors of neutrophil and RBC counts were approximately parallel to PC2. Non-operation group (n = 26), Sham-operation groups (n = 37 at 30 min, n = 21 at day 1, and n = 21 at day 4), and Thrombosis groups (n = 39 at 30 min, n = 13 at day 1, and n = 20 at day 4).

In contrast to the sham group samples, the thrombosis group samples (red color) were mostly located on the negative side of Principal Component (PC) 1, which corresponded to the positive direction of the monocyte vector and the negative direction of the platelet and lymphocyte vectors; the opposite results were obtained for the sham group. Separation increased progressively from 30 min to day 1 and 4, and it was better for PC1 than it was for PC2 (Fig. 4).

## Discussion

This is the first study to show that arterial thrombosis is associated with a) monocytosis and high MLR (post-thrombosis 30 min, 1 day, and 4 days), b) mild relative lymphopenia and thrombocytopenia (1 and 4 days), and c) high NLR and PLR (4 days) in a mouse model of carotid thrombosis. We suggest potential utility of these CBC parameters for predicting or detecting arterial thrombosis at an early phase, considering the lack of clinically available diagnostic tools for nascent thrombosis.

Monocytosis was previously thought to be a relatively non-specific finding that can be observed in inflammatory and stress-related conditions to be associated with neutrophilia. However, Swirski and Nahrendorf et al. revealed that monocytes have important pathophysiologic roles in acute vascular events such as myocardial infarction and stroke^7^. A recent study reported a role for monocytes, linking inflammation with arterial thrombosis^8^. Another recent study showed that MLR was better correlated with the severity of coronary lesion in patients with non-ST-elevation myocardial infarction (non-STEMI) than NLR was^9^. Moreover, MLR has been shown to be an independent predictor for in-hospital and long-term major adverse cardiac events, including cardiac death, cardiac arrest, cardiac rupture, cardiogenic shock, cardiogenic syncope, acute congestive heart failure, and malignant arrhythmic events during hospitalization^10^. The present animal study shows, for the first time, MLR elevation as well as monocytosis and mild relative lymphopenia, and intriguingly, with no significant change in neutrophil count, at an early phase of arterial thrombosis. This finding deserves further investigation in humans.

Recently, ischemic stroke-related stress was shown to activate both the sympathetic nervous system and the hypothalamic–pituitary–adrenal axis, resulting in high systemic levels of the stress hormone glucocorticoid^11^. Thus, cerebral ischemia dramatically changed the number and phenotype of circulating white blood cells 3 days after transient middle cerebral artery occlusion, resulting in increased myeloid cells and substantially fewer lymphocytes due to reduced B-cell progenitor proliferation and differentiation along with enhanced apoptosis in mouse bone marrow^12^. In that study, we detected increased cortisol levels and peripheral lymphopenia in patients 3 days after stroke, with a significant inverse correlation between cortisol concentrations and lymphocytes. Likewise, elevated cortisol-related low lymphocyte count is commonly found in acute coronary syndrome^12^. In the present animal study, thrombosed mice and sham-operated mice exhibited similar cortisol levels at all time-points; in both groups, cortisol levels were significantly but slightly higher only at the 30 min time-point when compared with non-operated mice. Thus, it is understandable that lymphocyte count increased to the level of non-operated mice by day 1 in sham operated mice. Increased cortisol levels could explain the temporal decrease in lymphocytes in the sham-operated mice at 30 min. However, given that the count in thrombosed mice decreased further by day 4 with normal cortisol levels, the mild lymphopenia induced by carotid thrombosis in this study may have resulted from factors other than increased cortisol levels. Moreover, there was no mortality and no post-operative anemia. Taken together, these results suggest a) a limited role of lymphocyte count by itself in terms of its potential utility regarding arterial thrombosis and b) the absence of substantial systemic stress or inflammation due to surgical modeling itself.

Both neutrophilia and lymphopenia can serve as components of an inflammatory or stress response in a variety of illnesses, including cardiovascular diseases. The number of circulating neutrophils depends on production vs. utilization. Neutrophil production is increased in response to inflammation; however, the neutrophil numbers may be variable (i.e., increased, decreased, or normal) in patients with inflammatory disease. Neutrophils could also shift from marginal to circulating pool by stress-related corticosteroids and catecholamines/epinephrine^13–15^. Likewise, acute inflammation can be accompanied by lymphocytopenia partly due to increased corticosteroid level, resulting in a) the migration of lymphocytes from blood into lymphoid tissues or to the inflammatory site, b) decreased lymphocyte production, or c) increased lymphocyte death^16^.

Because postoperative increases in cortisol levels may be transient and less pronounced, as shown in this study, the combination of elevated neutrophils and low levels of lymphocytes into a single composite marker of inflammation appears to be more discriminative in predicting cardiovascular manifestation and outcomes. Indeed, NLR has been shown to predict future vascular events^17–22^, coronary artery disease severity, and poor clinical outcomes such as increased incidence of heart failure and long-term mortality in patients with myocardial infarction^23^. Moreover, NLR could predict no reflow in patients with ST-elevation myocardial infarction (STEMI) undergoing primary coronary intervention^24^; a recent meta-analysis reported that thrombus burden is one of the most impacted risk factors of no-reflow phenomenon in STEMI^25^. Our animal study showed that acute carotid thrombosis was associated with a higher NLR at 30 min ~ 4 days, compared with non-operated mice. However, we found that the association of NLR with the arterial thrombosis remained significant only for post-thrombosis 4 days when considering the surgical modeling-related stress and inflammation by comparing with sham-operated mice. Since mild relative neutrophilia after carotid thrombosis is thought to be related to surgical modeling rather than thrombosis itself, the high NLR (in thrombosed vs. sham animals) at 4 days appears to be due more to lymphopenia.

PLR provides composite information about inflammation and hemostasis/thrombosis, and it could therefore better predict coronary atherothrombotic burden than either platelet or lymphocyte count alone. In line with this notion, PLR could independently predict coronary artery disease severity^26^, no-reflow development in patients with STEMI^27^, and in-hospital mortality in patients with STEMI^28^. Our animal study showed that PLR was about twofold higher 4 days after acute carotid arterial thrombosis, which may warrant further clinical investigation to study the potential roles of PLR as well as MNR and NLR to detect nascent thrombosis by performing CBC—a low-cost test—routinely and more often in subjects at a higher risk of atherothrombosis.

Unexpectedly, both platelet count and MPV were relatively low in thrombosed mice, although they were only slightly low and (mostly) within the normal range. Activated platelets aggregate together to form clots, which could decrease the platelet count. Recently, thrombin- or collagen-induced acute pulmonary thromboembolism was shown to be associated with thrombocytopenia^29,30^, suggesting that mild thrombocytopenia could possibly occur as a result of thrombus formation-related platelet consumption after FeCl_3_ application on the carotid artery. As a marker of platelet activity, an elevated MPV has been associated with acute myocardial infarction, restenosis following coronary angioplasty, and post-MI mortality^31^. However, a low MPV level has been found to be associated with inflammatory states in rheumatic patients^32^. Although speculative, given the arterial exposure to the corrosive agent in the animal model we used, it might also be helpful to consider low platelet production in the bone marrow (with or without systemic inflammation). To summarize, the low platelet count and low MPV found in our study might be attributable to platelet consumption, systemic inflammation, and/or low platelet production in the bone marrow. Alternatively, younger platelets with high MPV might have been consumed more in the clot and in the injured arterial wall. It has been proposed that 82% of platelet turnover in normal persons is due to senescence, and 18% (≈7100 platelets/μL/day) is due to the fixed requirement to maintain vascular integrity^33^. Further investigation is required to clarify whether or how low platelet count could be accompanied by low MPV in a mouse model of FeCl_3_-mediated arterial thrombosis.

Although our clarification of the hematologic response of the body to arterial thrombosis is an important set of findings, this study has several limitations. First, the alterations of the CBC-related parameters that we found may not necessarily be specific to arterial thrombosis. In a previous study^34^, neutrophil levels were found to be higher in a rat model of venous thrombosis that was induced by ligation of the inferior vena cava, compared with surgically naïve control rats. However, unlike our study on FeCl_3_-induced arterial thrombosis in mice, there were no significant differences in the levels of monocytes, lymphocytes, and platelets. Further hematological investigations should be conducted to compare arterial thrombosis with venous thrombosis or vasculitis in mice, preferably by using comparable modeling methods. Second, there are physiologic and immunologic differences between mice and humans. The most striking difference is the predominant WBC type, which is lymphocyte in mice vs. neutrophil in humans. Platelets also differ in terms of their number, size, and mRNA content^35^. These differences may affect the host response to arterial thrombosis, including the changes of CBC-related parameters. Therefore, our preclinical results must be confirmed in patients, although a) it is difficult to investigate if thrombosis per se, such as thrombosis before cerebral infarction, could alter CBC parameters in humans, and b) the animal model of FeCl_3_-mediated thrombosis, which is the most widely used in vivo model of in situ thrombosis, has proven its utility for bench-to-bedside translation such as accurate assessments of anti-thrombotic efficacy of clinically available anticoagulant or antiplatelet drugs and thrombolytic agents^36^. Third, additional experiments such as coagulation assays, platelet activity assays, and histological analysis of thrombus could have better validated the CBC-related parameters as a potential biomarker of arterial thrombosis. Fourth, we did not measure the occlusion time. Further investigations comparing the kinetics of post-thrombosis serial alterations in CBC-related parameters while considering the occlusion time would be an interesting follow-up study.

## Conclusion

Our study on serial changes in post-thrombosis CBC parameters provides the first preclinical evidence for the arterial thrombosis-related elevation of MLR, NLR, and PLR as well as monocytosis with mild relative lymphopenia but without neutrophilia. Further clinical studies are needed to investigate whether high levels of these composite CBC parameters precede acute cardiovascular events such as ischemic stroke or myocardial infarction. In addition to the potential clinical implications, the preclinical data will be useful for the planning and interpretation of vascular research using the mouse model of FeCl_3_-mediated thrombosis.

## Methods

### Animals and experimental groups

This study was approved by the Animal Ethics Committee at Preclinical Research Institute of Dongguk University Ilsan Hospital (#2019–10,191). All experimental procedures were performed in accordance with the National Institutes of Health guidelines. In addition, the study was carried out in compliance with the ARRIVE guidelines (https://arriveguidelines.org). Twelve-week-old male C57Bl/6 mice (total n = 228) were purchased from DBL Co. (Incheon, Korea) and fed ad libitum in a pathogen-free and climate-controlled environment that was maintained at 20 °C and 40 to 50% humidity, with 12 h of light per 24-h period. Two mice were excluded because of clotting during blood collection, and 226 mice in total were included in the final analysis.

Mice were anesthetized with an induction chamber using 2 ~ 2.5% isoflurane mixed with 30% oxygen (1.5 L/min), and in situ carotid thrombus was formed (n = 72) by an experienced researcher (J. Kim), as has been previously reported^37,38^, by applying a strip of 1 × 1 mm^2^ filter paper (grade 42; Whatman, Oxon, UK) soaked in 4 μL 10% FeCl_3_ to the left common carotid artery (CCA) for 10 min after exposing the vessel by a midline neck incision and dissection of the perivascular tissue. We previously demonstrated that similar-sized carotid thrombi can be consistently generated at the hands of a skillful researcher^39^. In sham-operated animals (n = 79), the left CCA was exposed to saline-soaked filter paper for 10 min. A CBC test with WBC differential (ADVIA2120i, Siemens, Munich, Germany) was performed at one of the following time-points: 30 min, 1 day, and 4 days after thrombosis or sham operation. The time-points were selected to cover the hyperacute (30 min) and acute (1 and 4 days) period of ischemic stroke^40^, which is mostly attributable to arterial thromboembolism. Moreover, non-operated mice (n = 26) also underwent a CBC test with WBC differential. To date, there has been no publication on arterial thrombosis-related changes in CBC parameters in mice. Therefore, the sample sizes were empirically determined as being mostly 20 or higher after considering the results of an aforementioned paper^34^ that reported venous thrombosis-related changes in CBC and WBC differential parameters in rats, where the sample size of 18 per group was sufficient to show significant differences in blood neutrophil levels between the venous thrombosis group and the control group.

In a different set of animals (n = 49) with/without thrombosis or sham operation, cortisol levels were measured using an electrochemiluminescence immunoassay (Cobas 8000, Roche, Mannheim, Germany) to account for surgical stress-related CBC/WBC differential alterations. Blood (~ 500 μl) was drawn from the retro-orbital plexus and collected in Vacutainer® EDTA tubes (BD KOREA Co. Ltd., Seoul, South Korea) in all mice.

Animals were randomly assigned to the experimental groups by H.J. Jang, and the operator (J. Kim) was blinded to the allocation throughout the experiment. After the induction of inhalation anesthesia and blood sampling, mice were euthanized through cervical dislocation.

### Statistical analyses

Data are presented as the mean ± standard deviation or median (interquartile range). Comparisons between experimental groups were performed using one-way ANOVA and Bonferroni post-hoc tests. A p value of < 0.05 was considered to be statistically significant. When the normality assumption was not met (as determined according to the Shapiro–Wilk test), comparisons were made using Kruskal–Wallis tests with Dunn’s post-hoc tests. Principal component analysis (PCA)^41^ was performed to observe thrombosis (vs. sham operation)-related data distribution in representative low-dimensional spaces while accounting for multiple CBC parameters (neutrophil count, lymphocyte count, monocyte count, platelet count, and RBC count). Briefly, for each time-point, we identified the first three principal components (PC1, PC2, and PC3) in descending order of explanatory power for variance. The loading weights on the first three PCs were estimated for each CBC parameter to investigate how much weight each original parameter contributes to the corresponding principal component. PCA biplots^42^ were generated for each time-point to visually assess data grouping by using their projections onto the first two PCs. Moreover, loading vectors were displayed in the biplots to represent how each CBC parameter contributed to the variability.

## Data Availability

The datasets used and/or analyzed during the current study are available from the corresponding author on reasonable request.
